# Cryptotanshinone induces ROS-mediated apoptosis in human gastric cancer cells

**DOI:** 10.18632/oncotarget.23267

**Published:** 2017-12-15

**Authors:** Chang Liu, Hu-Nan Sun, Ying-Hua Luo, Xian-Ji Piao, Dan-Dan Wu, Ling-Qi Meng, Yue Wang, Yi Zhang, Jia-Ru Wang, Hao Wang, Wan-Ting Xu, Jin-Qian Li, Yang Liu, Yi-Qin Wu, Ying-Hao Han, Gui-Nan Shen, Mei-Hua Jin, Yan-Qing Zang, Jing-Chun Li, Nan-Zhu Fang, Yu-Dong Cui, Cheng-Hao Jin

**Affiliations:** ^1^ Department of Biochemistry and Molecular Biology, College of Life Science & Technology, Heilongjiang Bayi Agricultural University, Daqing 163319, China; ^2^ College of Animal Science & Veterinary Medicine, Heilongjiang Bayi Agricultural University, Daqing 163319, China; ^3^ Department of Gynaecology and Obstetrics, The Fifth Affiliated Hospital of Harbin Medical University, Daqing 163316, China; ^4^ College of Food Science & Technology, Heilongjiang Bayi Agricultural University, Daqing 163319, China; ^5^ Department of Animal Science, College of Agriculture, Yanbian University, Gongyuan-jie, Yanji 133002, China

**Keywords:** cryptotanshinone, gastric cancer, apoptosis, cell cycle arrest, ROS

## Abstract

Cryptotanshinone (CT), isolated from the plant *Salvia miltiorrhiza Bunge*, has been reported to have potential anticancer effects on human prostate and breast cancer cells. However, the mechanisms of action of CT on gastric cancer (GC) cells are not well understood. Here we investigated the antitumor effects of CT on GC cells and its possible molecular mechanism. We found CT suppressed viability of twelve GC cell lines in a dose-dependent manner. CT induced cell cycle arrest at the G2/M phase and mitochondrial apoptosis accompanying the accumulation of reactive oxygen species (ROS). Pretreatment with ROS inhibitor N-acetyl-L-cysteine (NAC) blocked CT-induced apoptosis. CT increased p-JNK and p-p38, and decreased p-ERK and p-STAT3 protein expression, these effects were prevented by NAC. Furthermore, a xenograft assay showed that CT significantly inhibited MKN-45 cell-induced tumor growth *in vivo* by increasing expression of pro-apoptotic proteins (p-JNK, p-38 and cleaved-caspase-3) and reducing expression of anti-apoptotic proteins (p-ERK and p-STAT3) without adverse effects on nude mice weight. In conclusion, CT induced apoptosis and cell cycle arrest in GC cells via ROS-mediated MAPK and AKT signaling pathways, and this CT may be a useful compound for the developing anticancer agents for GC.

## INTRODUCTION

Gastric cancer (GC) is the fourth most common cancer and the second leading cause of cancer death worldwide [1]. At present, the main treatments for GC include surgical resection, chemotherapy and radiotherapy [2]. However, these treatments have only modest efficacy for advanced or metastatic GC and they have a range of side effects, such as hair and hearing loss and kidney damage. Therefore, it is necessary to develop novel chemotherapies that are highly effective and less toxic.

Mitogen-activated protein kinase (MAPK) family, which mainly consists of extracellular-signal-regulated kinase (ERK), c-jun N-terminal kinase (JNK), and p38 are mediators of intracellular signals involved in the regulation of processes including cell growth and apoptosis [3–5]. ERK, JNK and p38 are all serine/threonine kinases that are directed by a proline residue. Along with the pathways in which these three MAP kinases are activated share similarity by extracellular or intracellular stimuli, such as reactive oxygen species (ROS) [6]. ROS such as oxygen ions, superoxide and hydrogen peroxide (H_2_O_2_) or hydroxyl radicals which mediate intracellular signaling cascades may trigger redox signaling pathways, such as oxidative stress, apoptosis, and cell cycle arrest [7, 8]. According to these recent studies, many chempreventive and chemotherapeutic agents can increase the generation of ROS and induced cancer cell apoptosis via mitochondrial-dependent signaling pathway. In addition, distribution of the mitochondrial transmembrane potential is considered to be one of the apoptotic processes induced by chemotherapeutic drugs.

Cryptotanshinone (CT) is a major active component of *Salvia miltiorrhiza Bunge* (Danshen) [9], which has been applied to treat cardiovascular diseases, Alzheimer’s disease and inflammation [10–12]. CT may have anticancer activity via the inhibition of proliferation of various tumor cells or via induction of apoptosis in MCF7 breast cancer cells, A2058, A375 and A875 melanoma cells, BxPC-3 pancreatic cancer cells and A549 lung cancer cells [13–16]. Thus, we seek to understand whether CT induces apoptosis and inhibits tumor growth in GC cells, thereby blocking GC tumors.

In the present study, we investigated antitumor activities effect of CT on GC cells and attempted to explain underlying mechanisms. Our results showed that CT inhibited growth of AGS, MKN-28, MKN-45 and 9 other GC cells. CT also suppressed tumors derived from MKN-45 cells inoculated in nude mice. The antitumor effect of CT may be attributed to accumulated ROS leading to p-JNK and p-p38 increasing and p-ERK, p-Akt and p-STAT3 decreasing, finally induced apoptosis and G2/M phase arrest. These data suggest that CT is worthy of further study for the treatment of GC.

## RESULTS

### CT inhibited proliferation and induced apoptosis of GC cells

**Figure 1:**
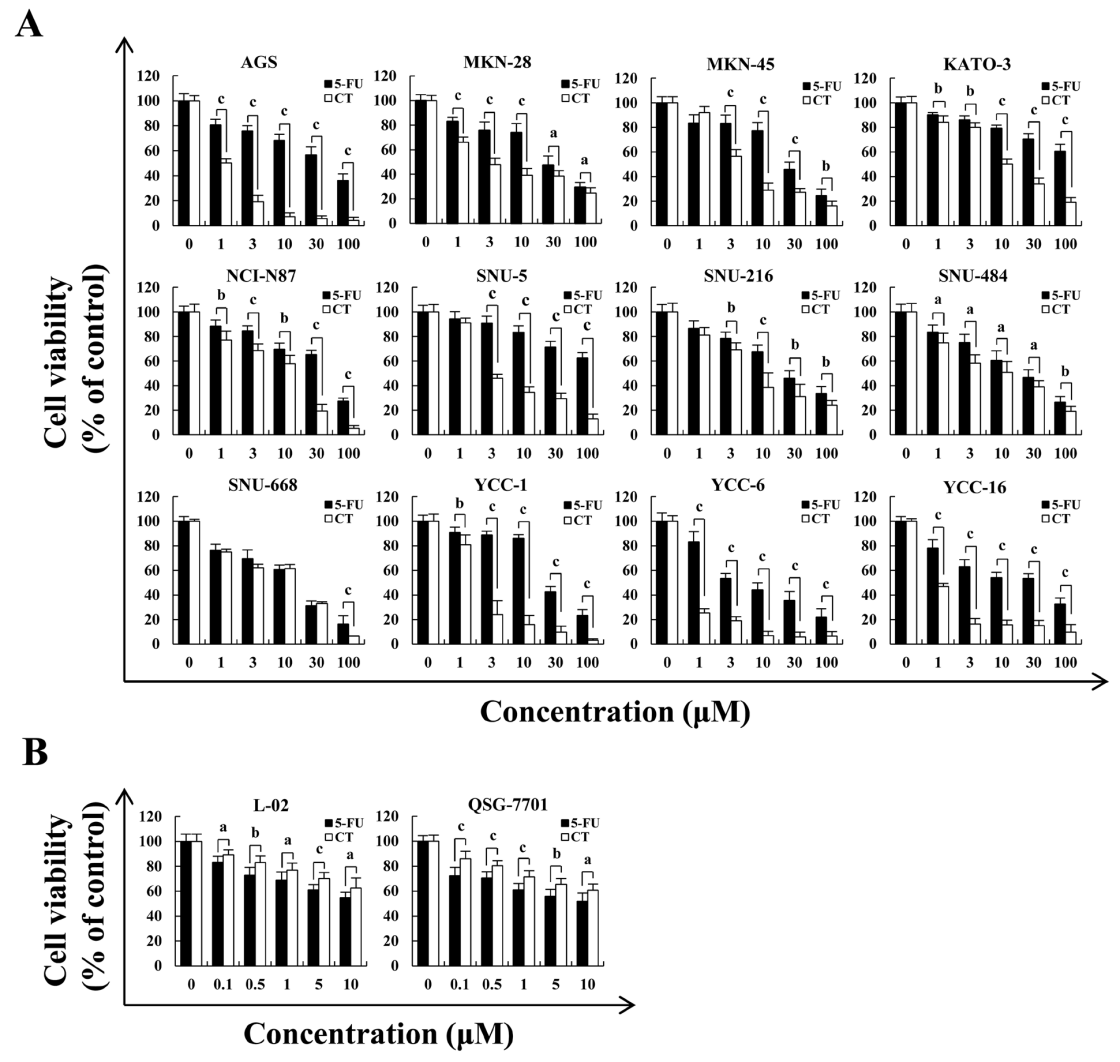
Cytotoxic effects of CT on multiple GC cell lines **(A)** AGS, MKN-28, MKN-45, KATO-3, NCI-N87, SNU-5, SNU-216, SNU-484, SNU-668, YCC-1, YCC-6 and YCC-16 cells were treated with 1, 3, 10, 30 and 100 μM of 5-FU or CT for 24 h, then cell viability was measured by MTT assay. **(B)** Human liver L-02 and QSG-7701 cells were treated with 0.1, 0.5, 1, 5 and 10 μM of 5-FU or CT for 24 h, then cell viability was measured by MTT assay. Error bars indicate means ± SD of three independent experiments (^a^*p*<0.05, ^b^*p*<0.01, ^c^*p*<0.001 indicated significant differences).

**Figure 2:**
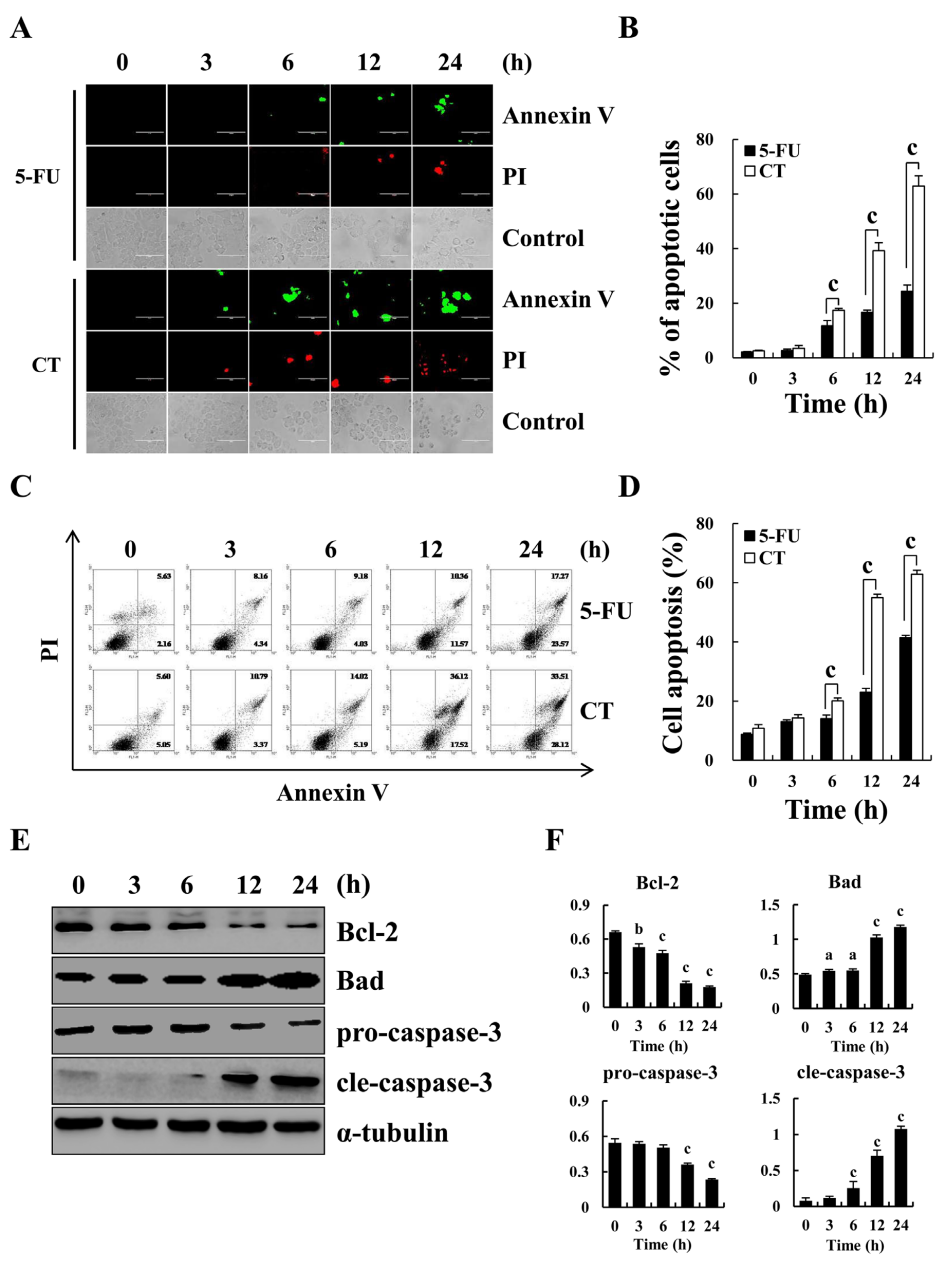
Apoptosis induced by CT in GC cells **(A)** Induction of apoptosis in human gastric cancer AGS cells by Annexin V-FITC/PI double staining and observed under a fluorescence microscope after treatment with 10 μM CT for 3, 6, 12 and 24 h, respectively. Scale bar 100 μm. **(B)** Quantification of fluorescent intensity. **(C)** AGS cells treated with 10 μM CT for 3, 6, 12 and 24 h, respectively. Apoptotic cells were stained with Annexin V-FITC and PI, and measured using flow cytometry **(D)** Quantification of apoptotic cells in (C). **(E)** Apoptosis-related protein Bcl-2, Bad, pro-caspase-3 and cleaved-caspase-3 was measured using western blot, respectively. **(F)** Quantification of the band density was analyzed by ImageJ software (^a^*p*<0.05, ^b^*p*<0.01, ^c^*p*<0.001 indicated significant differences).

Figure 1 showed that CT treatment dose-dependently decreased GC cell viability and the cytotoxic effects of CT was less than those of 5-FU in normal liver L-02 and QSG-7701 cells. IC_50_ values for CT and 5-FU for each cell line appear in Table 1. AGS, MKN-28 and MKN-45 cells were more sensitive to CT compared (Figure 1A and Table 1). Therefore, we used AGS, MKN-28, and MKN-45 cells. Figure 2A shows that compared with controls, treatment with CT for 24 h caused cell shrinkage and membrane blebbing. Annexin V-FITC and PI staining showed increased fluorescence with increased treatment time with CT in AGS cells. This was significantly different that fluorescence in 5-FU treated cells (Figure 2B). Flow cytometry (Figure 2C and 2D) showed increased apoptosis in a time-dependent manner. Finally, CT increased Bad and cleaved-caspase-3 expression but decreased pro-caspase-3 and Bcl-2 protein expression in a time dependent manner (Figure 2E and 2F). Thus, cytotoxic effects of CT on GC cells are attributable to mitochondrial-mediated apoptosis.

**Table 1:** IC_50_ value of 5-FU and CT in 12 GC cell lines

	5-FU (μM)	CT (μM)
AGS	31.86±0.04	0.95±0.05
MKN-28	28.05±0.05	2.11±0.03
MKN-45	27.48±0.04	4.63±0.04
KATO-3	>100	10.06±0.03
NCI-N87	57.63±0.04	11.53±0.03
SNU-5	>100	2.27±0.06
SNU-216	26.22±0.05	7.41±0.04
SNU-484	25.21±0.04	11.5±0.05
SNU-668	17.47±0.04	19.89±0.04
YCC-1	26.67±0.04	2.13±0.05
YCC-6	5.78±0.05	0.67±0.03
YCC-16	41.56±0.03	0.81±0.04

### CT triggered cell cycle arrest at G2/M phase

**Figure 3:**
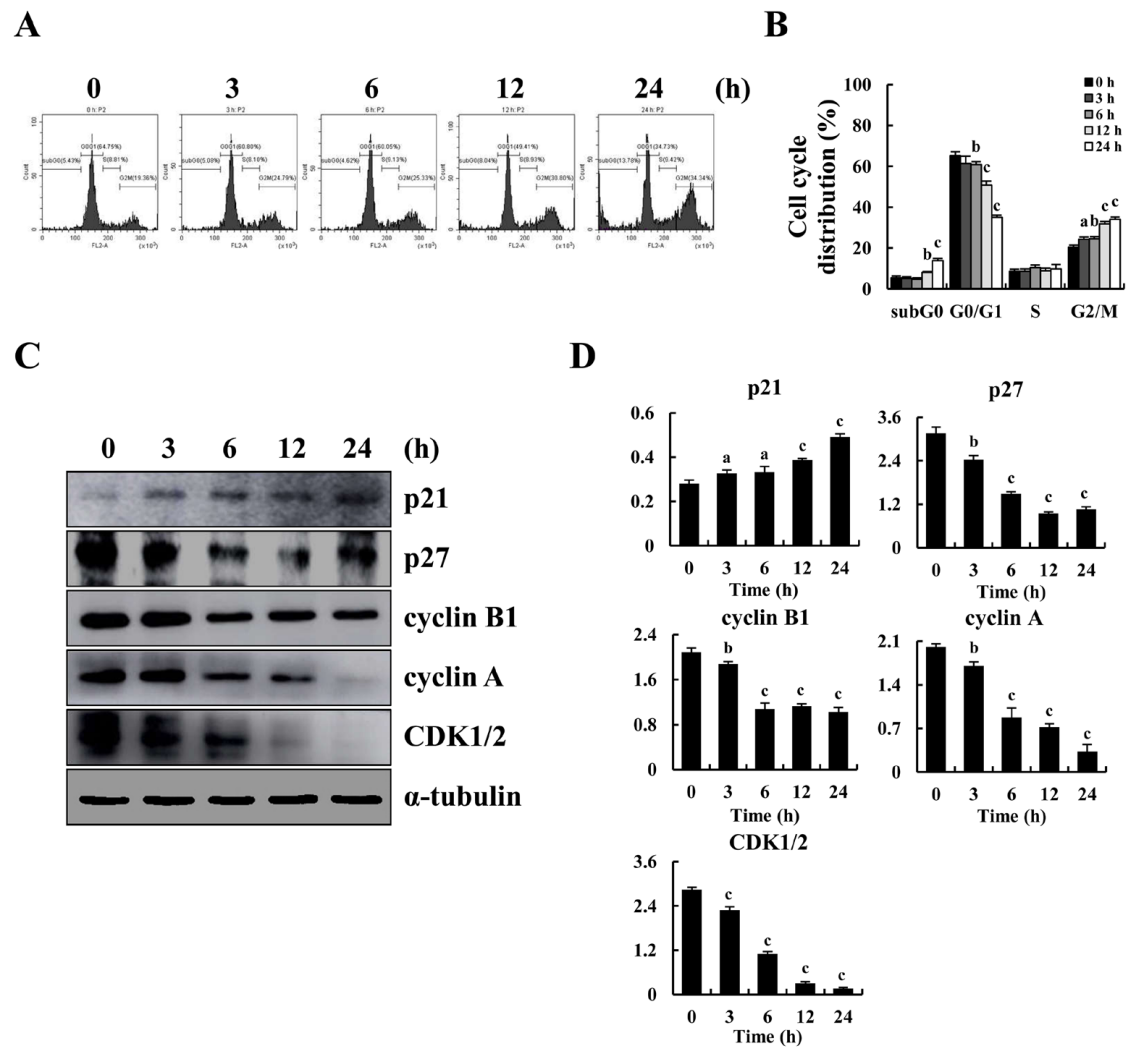
G2/M cycle arrest induced by CT and altered expression of cell cycle-regulatory protein in MKN-28 cells **(A)** Cell cycle distribution of MKN-28 cells monitored by flow cytometer after treatment with 10 μM CT for 3, 6, 12 and 24 h, respectively. **(B)** Representative histograms from flow cytometry in human gastric cancer MKN-28 cells treated with CT. Assays performed in triplicate. **(C)** Expression of G2/M cell cycle proteins p21, p27, cyclin B1, cyclin A and CDK1/2 were assayed by western blot after treatment with 10 μM CT for 3, 6, 12 and 24 h, respectively. α-tubulin was used as internal control. **(D)** Western blot results from (C) was calculated and represented as percent of control (^a^*p*<0.05, ^b^*p*<0.01, ^c^*p*<0.001 indicated significant differences).

To explore whether cell cycle arrest led to apoptosis, MKN-28 cells were stained with PI and the cell cycle was assayed. Figure 3A shows that after treatment with 10 μM CT at the indicated time points, G2/M phase arrest occurred in a time-dependent manner and that fewer cells had G0/G1 and S DNA content. Next, western blot results showed that CT treatment decreased expression levels of p27, CDK1/2, cyclinB1 and cyclin A, and increased expression level of p21 consistent with G2/M cell cycle arrest (Figure 3C and 3D).

### CT induced apoptosis and cell cycle arrest via MAPK and AKT signaling pathway

**Figure 4:**
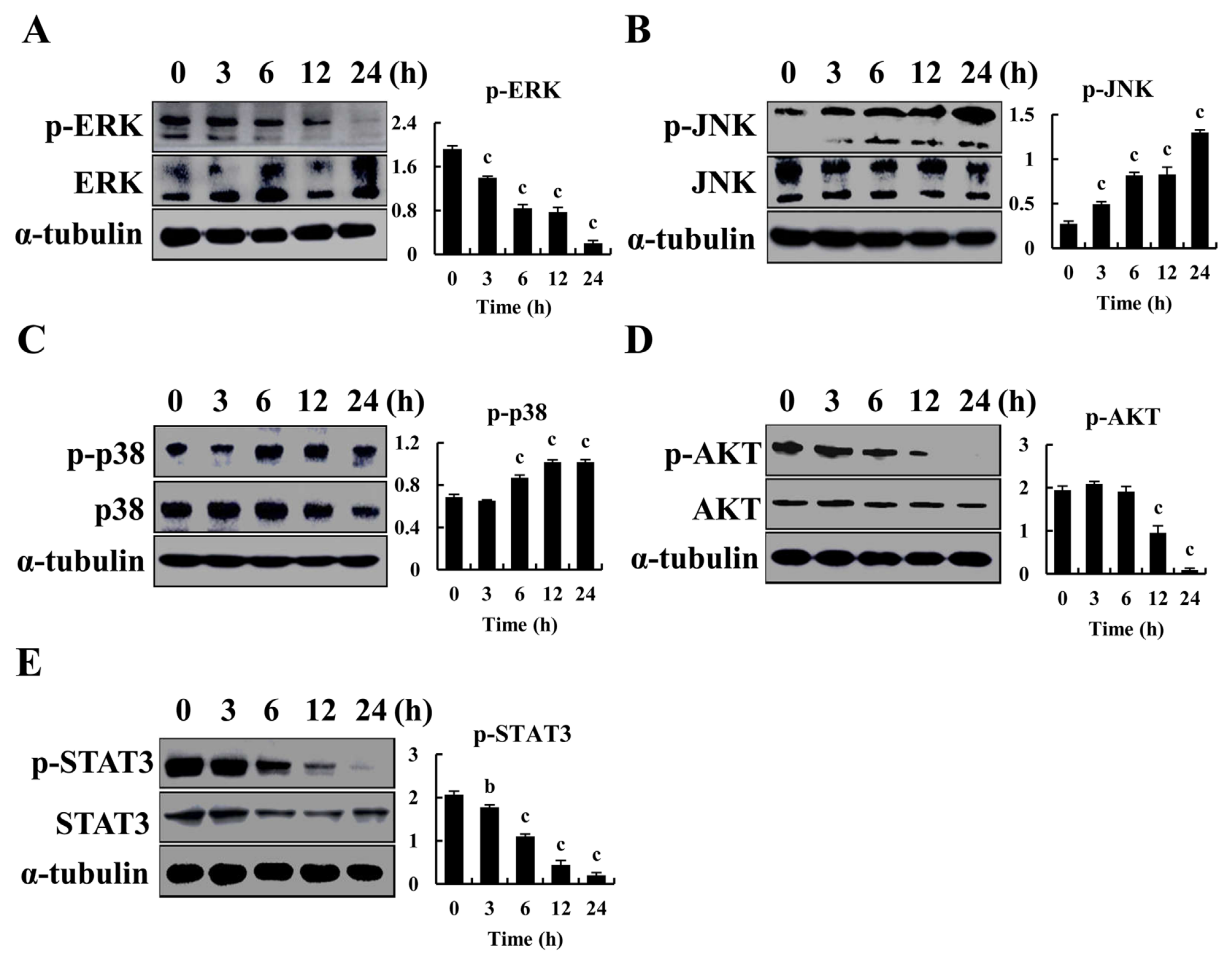
Regulates MAPK, AKT and STAT3 signaling pathways by CT in GC cells **(A-E)** p-ERK, p-JNK, p-p38, p-AKT and p-STAT3 was measured by western blot after treatment with 10 μM CT for 3, 6, 12 and 24 h, respectively in AGS cells (^a^*p*<0.05, ^b^*p*<0.01, ^c^*p*<0.001 indicated significant differences).

Western blot was used to confirm that expression of MAPK, AKT and STAT3 (Figure 4A-4E) significantly decreased, and p-JNK and p-p38 expression was increased in AGS cells. Thus, CT induced apoptosis and cell cycle arrest through MAPK and AKT signaling pathways.

### CT induced ROS accumulation which participated in apoptosis of MKN-28 cells

**Figure 5:**
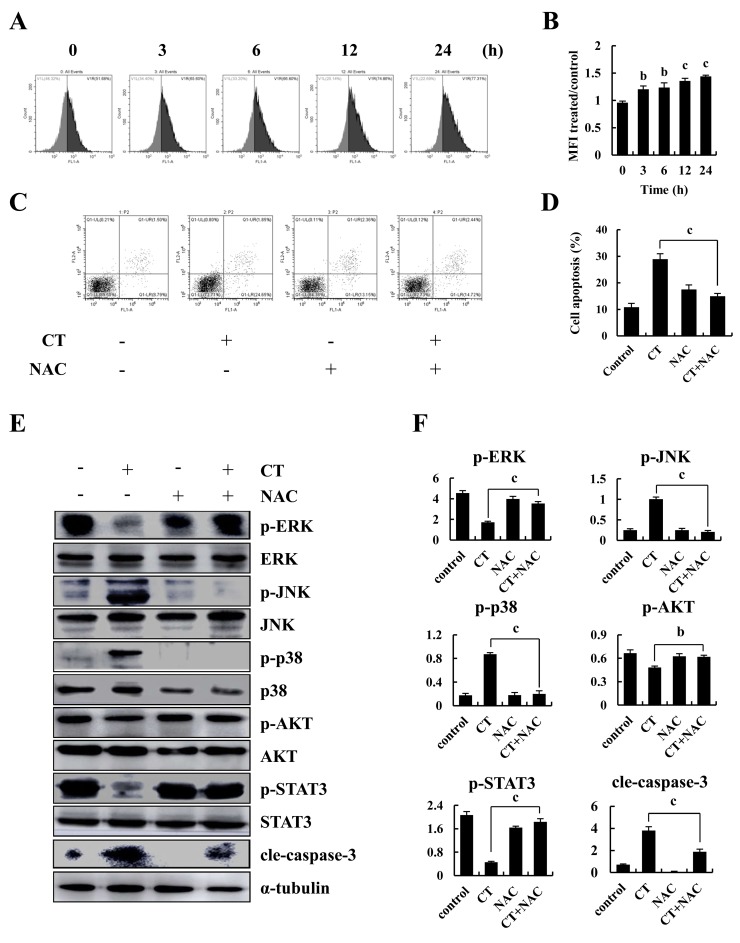
Intracellular ROS generation and apoptosis induced by CT in human gastric cancer cell **(A-B)** Intracellular ROS generation induced by CT was measured in MKN-28 cells using DCFH-DA (10 μM) and flow cytometry. **(C-D)** Blocking ROS generation abolished CT-induced apoptosis. MKN-28 cells were pre-incubated with/without 5 mM of NAC for 30 min before exposure to CT (10 μM) for 24 h. Apoptosis was measured by flow cytometry. **(E-F)** MKN-28 cells were pretreated with/without 5 mM of NAC for 30 min before exposure to CT (10 μM) for 24 h. p-ERK, p-JNK, p-p38, p-AKT and p-STAT3 protein expression was measured by western blot (^a^*p*<0.05, ^b^*p*<0.01, ^c^*p*<0.001 indicated significant differences).

Figures 5A and 5B show that CT significantly increased fluorescent intensity of MKN-28 cells in time-dependent manner, indicating that CT potentiated elevation of intracellular ROS. Also, pretreatment with NAC significantly inhibited CT-induced increased apoptosis (Figure 5C and 5D). NAC blocked CT-induced decreased expression of p-ERK, p-AKT and p-STAT3 and increased expression of p-JNK and p-p38 and cleaved-caspase-3 (Figure 5E and 5F). Therefore, CT-induced ROS generation is an early event that induces MAPK and Akt activation and triggers mitochondrial apoptotic pathways in MKN-28 cells.

### CT suppressed tumor growth in mouse xenograft models

**Figure 6:**
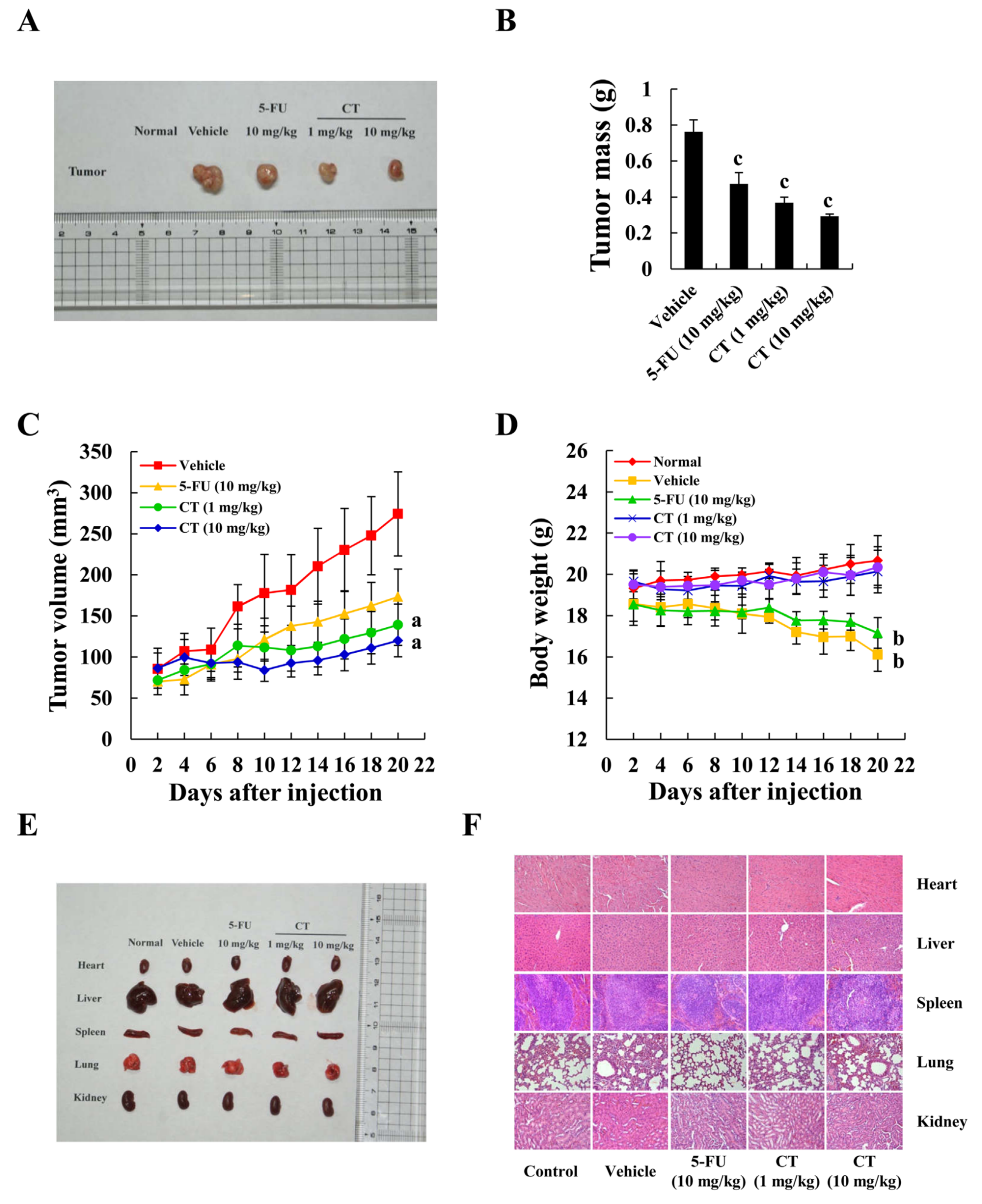
Inhibition of gastric cancer tumor xenograft growth by CT MKN-45 cells in equal amount were inoculated subcutaneously into the flanks nude mice. **(A)** Tumors were removed and images were captured. **(B)** Tumor weight after injection. **(C)** Tumor size was recorded once every two days using Vernier caliper measurements and calculated as [(length×width^2^)/2]. **(D)** Animal weight was recorded once every two days. **(E)** Organ images after injection. **(F)** Structural changes according to H&E staining under a light microscope. Scale bar 100 μm (^a^*p*<0.05, ^b^*p*<0.01, ^c^*p*<0.001 indicated significant differences).

Figure 6A and 6B show that tumors after CT treatment were smaller and fewer than in controls. On days 12–20 day, tumors were suppressed (Figure 6C). 5-FU inhibited tumor growth, but mouse weight decreased, in contrast to CT treatment which caused weight to increase (Figure 6D). During treatment (21 days), no mouse appeared to have toxic side effects and hematological assays confirmed no changes in experimental animals (See Table 2). No morphological changes were observed in organs of tumor-bearing mice treated with CT according to H&E staining (Figure 6E and 6F).

**Table 2:** Hematological parameters of nude mice bearing MKN-45 cell xenograft tumors

Hematological parameters	Normal	Vehicle	5-FU 10 mg/kg	CT		Standard
				1 mg/kg	10 mg/kg	
White blood cells (10^9^/L)	9.98±1.54	9.48±3.14	10.75±1.78	10.29±1.11	10.82±1.39	4.0-12.0
Lymphocytes percentage (%)	65.01±17.02	50±17.74	69±10.6	73.65±7.2	70.85±13.55	54.0-85.0
Middle cells percentage (%)	1.59±0.77	1.43±0.82	1.54±0.53	1.19±0.47	1.65±0.07	0.0-20.0
Granulocyte percentage (%)	33.4±6.74	27.7±8.47	19.3±5.78	16.17±7.25	22.5±3.48	12.0-44.0
Lymphocytes (10^9^/L)	6.72±1.58	6.46±2.1	7.75±0.78	5.09±1.05	5.46±1.59	3.0-8.5
Middle cells (10^9^/L)	0.15±0.12	0.11±0.06	0.09±0.04	0.02±0.05	0.07±0.01	0.0-2.0
Granulocyte (10^9^/L)	2.12±1.58	3.33±1.65	2.86±1.58	4.19±1.31	2.27±2.23	0.7-5.0
Red blood cells (10^12^/L)	10.46±0.13	10.6±0.08	10.38±0.20	9.89±1.21	9.06±1.04	7.0-12.5
Hemoglobin (g/L)	152.57±14.16	161.25±3.77	159.45±5.26	170.15±2.99	149±15.25	100-190
Mean corpuscular volume (fL)	57.57±0.53	56.25±0.5	56.55±0.52	56.38±0.65	55.5±0.71	51.0-65.0
Hematokrit (L/L)	0.5±0.01	0.49±0.03	0.48±0.01	0.51±0.01	0.46±0.05	0.35-0.55
Mean corpuscular hemoglobin (Pg)	18.1±5.57	19.8±1.27	20.01±0.36	18.93±1.74	18±1.41	12.0-30.0
Mean corpuscular hemoglobin concentration (g/L)	314.86±6.89	285.75±3.86	295±6.93	270.62±13.18	301.5±10.51	230-330
Red blood cell distribution width_SD (fL)	41.4±0.27	40.63±0.39	40.85±0.41	40.3±1.19	39.15±2.05	15.0-55.0
Red blood cell distribution width_CV (%)	21.83±0.08	21.93±0.13	21.9±0.09	21.68±0.43	21.25±0.78	12.0-30.0
Blood platelet (10^9^/L)	186.29±34.43	144.25±29.96	218.91±21.02	214.38±47.93	200.5±54.45	100-300
Mean platelet volume (fL)	6.67±0.7	6.95±0.49	7.25±0.33	7.1±0.81	7.2±0.57	6.1-12.0
Thrombocytocrit (L/L)	0.12±0.04	0.1±0.03	0.16±0.02	0.15±0.04	0.14±0.03	0.10-0.60
Platelet distribution width (%)	15.89±0.37	15.15±0.21	15.46±0.24	15.67±0.35	15.14±0.37	1.0-30.0

Note: Blood samples were taken after CT or 5-FU treatment for 20 days.

### CT regulated the expression of MAPK, STAT3 and cleaved-caspase-3 in xenograft mice

**Figure 7:**
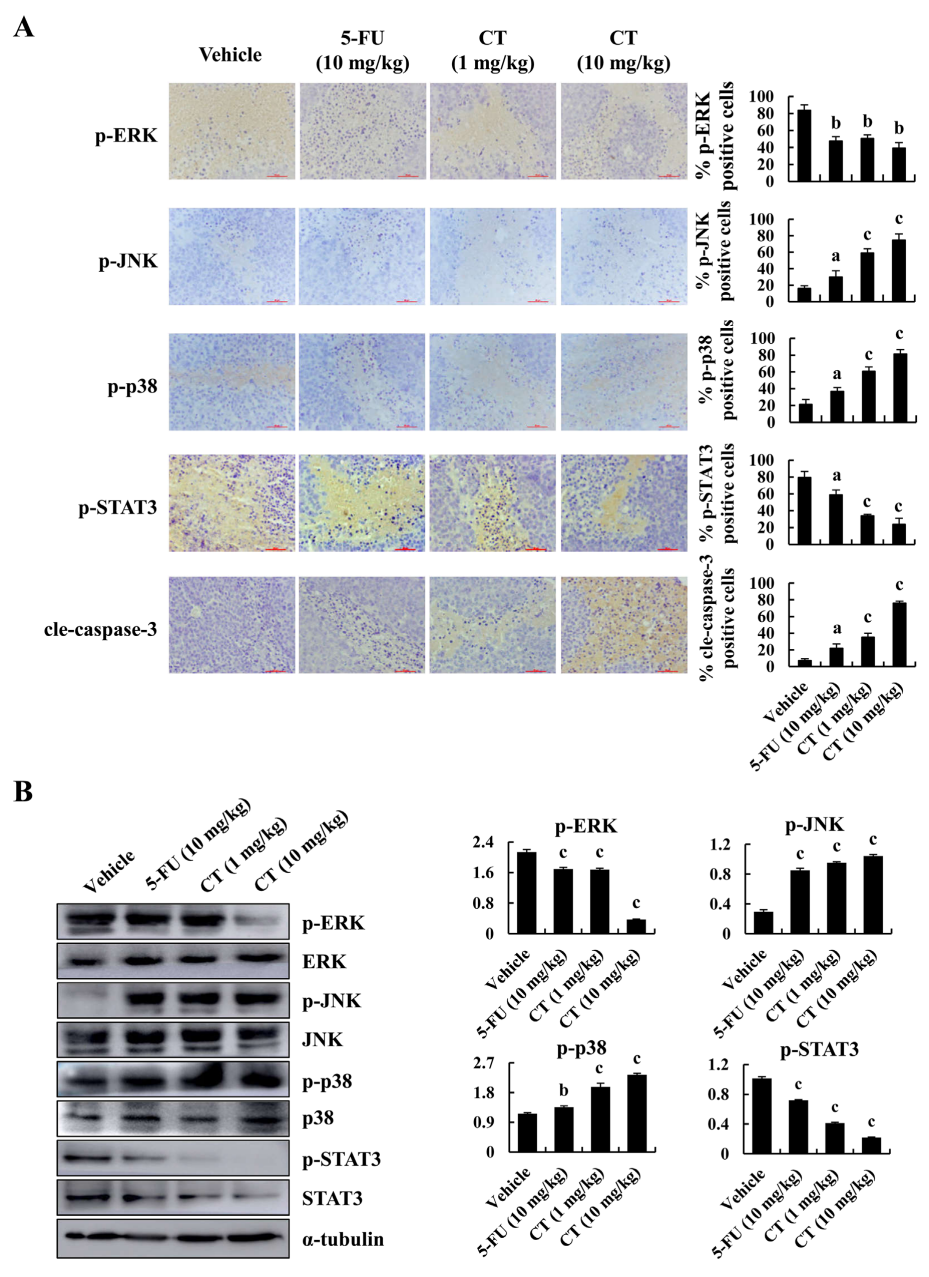
Immunohistochemical detection of key molecules in MAPK signaling pathways in xenograft tumor tissues **(A)** p-ERK p-JNK, p-p38, p-STAT3 and cleaved-caspase-3 expression in tumor tissues was measured by immunohistochemistry under a light microscope. Scale bar 50 μm (^a^*p*<0.05, ^b^*p*<0.01, ^c^*p*<0.001 indicated significant differences). **(B)** p-ERK p-JNK, p-p38 and p-STAT3 expression in tumor tissues was measured by western blot (^a^*p*<0.05, ^b^*p*<0.01, ^c^*p*<0.001 indicated significant differences).

CT induced apoptosis and inhibited tumor growth in GC cells. The immunohistochemistry staining result showed that expression levels of p-JNK, p-p38 and cleaved-caspase-3 were increased, whereas the expression levels of p-ERK and p-STAT3 were decreased in CT-treated xenograft tissues (Figure 7A). The western blot analyses of protein lysates from tumor tissue showed the similar results with IHC (Figure 7B). Taken together, these data demonstrate that CT has potent *in vivo* antitumor activity via MAPKs and STAT3 signaling pathway.

## DISCUSSION

Cryptotanshinone, tanshinones I, IIA, IIB and dihydrotanshinone are the most abundant constituents of the root of S. miltiorrhiza. Most studies have focused on the antioxidant and anti-inflammatory effect of tanshinones I and IIA, which may have anti-cancer effects [17, 18]. Recently, studies reported that CT could inhibit HL-60 human leukemic cell viability [19] and we report here that CT significantly inhibited the viability of AGS and other 11 GC cell lines (Figure 1). AGS, MKN-28 and MKN-45 cells were more sensitive than other gastric cancer cell lines. To identify how this occurs, we studied cell cycle distribution, cell cycle checkpoint protein expression, MAPK pathways, and the induction of apoptosis after treatment with CT on AGS, MKN-28 and MKN-45, respectively.

Apoptosis occurs chiefly through the extrinsic pathway (death receptor pathway) and the intrinsic pathway (mitochondrial pathway) [20]. In the extrinsic pathway, caspase-8 is activated, whereas caspase-9 is involved in the intrinsic pathway [21–23]. Changes in expression of pro-apoptotic protein (Bad) versus anti-apoptotic proteins (Bcl-2) activate the intrinsic apoptotic pathway [24]. It has been reported that CT can increase the expression levels of cleaved-caspase-3 and pro-apoptotic protein Bax while decrease Bcl-2 via the ROS-mitochondrial apoptotic pathway, and arrest the cell cycle at the G2/M phase in A375 melanoma cells [15]. Our data indicated that CT increased apoptosis in a time-dependent manner. On the molecular level, CT administration promoted cleaved-caspase-3 and expression of Bad and pro-caspase-3 and Bcl-2 were significantly decreased in a time-dependent manner after treatment with CT. Thus, CT may inhibit GC cell growth by inducing mitochondrial-mediated apoptosis.

Another main regulatory mechanism to control cell growth and induced cell apoptosis is through cell cycle control and several cytotoxic agents that arrest the cell cycle are currently used as antitumor drugs [25, 26]. In eukaryotes, the cell cycle is regulated by cyclins and cyclin-dependent kinases (CDKs) reduced activity of CDK1/2 and cyclinB1 is the hallmark of cell cycle arrest at the G2/M phase [27, 28]. Additionally, Akt kinase and signal transducer and activator of transcription (STAT3) contribute to cell survival, proliferation and cell cycle regulation [29, 30]. It has been reported that apoptosis is protected by Akt activity during the G2/M transition and it is necessary for effective entry to mitosis during unperturbed cell cycles [31]. STAT3 induces G2/M cycle arrest via regulating its downstream targets including p27, p21, cyclin B1 and so on [32–34]. Our results showed that CT reduced activation of Akt and STAT3 (Figure 3C and 3D & Figure 4D and 4E). Consequently, a significant accumulation occurred at the G2/M phase in MKN-28 cells after CT treatment, and this was associated with a significant decrease in cells at the G2/M phase.

Low ROS is important for cellular function and survival signaling and excessive ROS-elicited oxidative stress leads to cell death via apoptosis. Accumulated evidence indicates that chemotherapeutic agents can induce apoptosis via ROS production in various cancer cells [35]. Numerous anti-cancer drugs such as capsaicin, Khz-cp and apigenin could increase ROS production and induce cancer cells apoptosis by activating NADPH oxidase ROS catalytic subunit [36–38]. It has been reported that Nrf2 (Nrythroid-derived 2) activity could affect the function of two different sources of mitochondrial ROS production and production of ROS via NADPH oxidase [39]. Whether CT-induced gastric cancer cells apoptosis which caused by NADPH oxidase increasing the generation of ROS or the ROS production by mitochondria need to further study. Our result showed that CT-induced apoptosis was accompanied by ROS accumulation (Figure 5A and 5B), but that NAC reversed CT-induced apoptosis, indicating that apoptosis induced by CT was ROS-independent (Figure 5C and 5D).

The MAPK family has crucial roles in maintenance of cell survival and induction of apoptosis. In most cancer cells, ERK activated by growth factors is associated with cell proliferation. In contrast, JNK and p38 mediate cellular responses to stress and involved in proliferation, differentiation or apoptosis depending on the cell type and the stimuli. It has been reported that CT can activate p38/JNK and inhibit ERK1/2 via generation of ROS, leading to caspase-independent cell death in Rh30, DU145, and MCF-7 cells [40]. We report that CT induced phosphorylation of p38, JNK, and decreased phosphorylation of ERK in AGS cells in a time-dependent manner. NAC could also rescue MAPK activation. Thus, CT induces ROS-mediated MAPK activation and leads to apoptosis in GC cells.

In the previous study, we established AGS, MKN-28 and MKN-45 gastric cancer model of nude mice. But the tumor formation rate of the AGS and MKN-28 cells was lower than MKN-45 cells. Therefore, we choose MKN-45 cell to make a xenograft model. Our nude mice model bearing MKN-45 tumors showed that CT suppressed tumor growth significantly (Figure 6A-6C). Compared with controls, CT did not influence body weight (Figure 6D) and there was no hematological (Table 2) or organ toxicity (Figure 6E and 6F). Therefore CT had notable antitumor activity and was safe for this mouse model.

**Figure 8:**
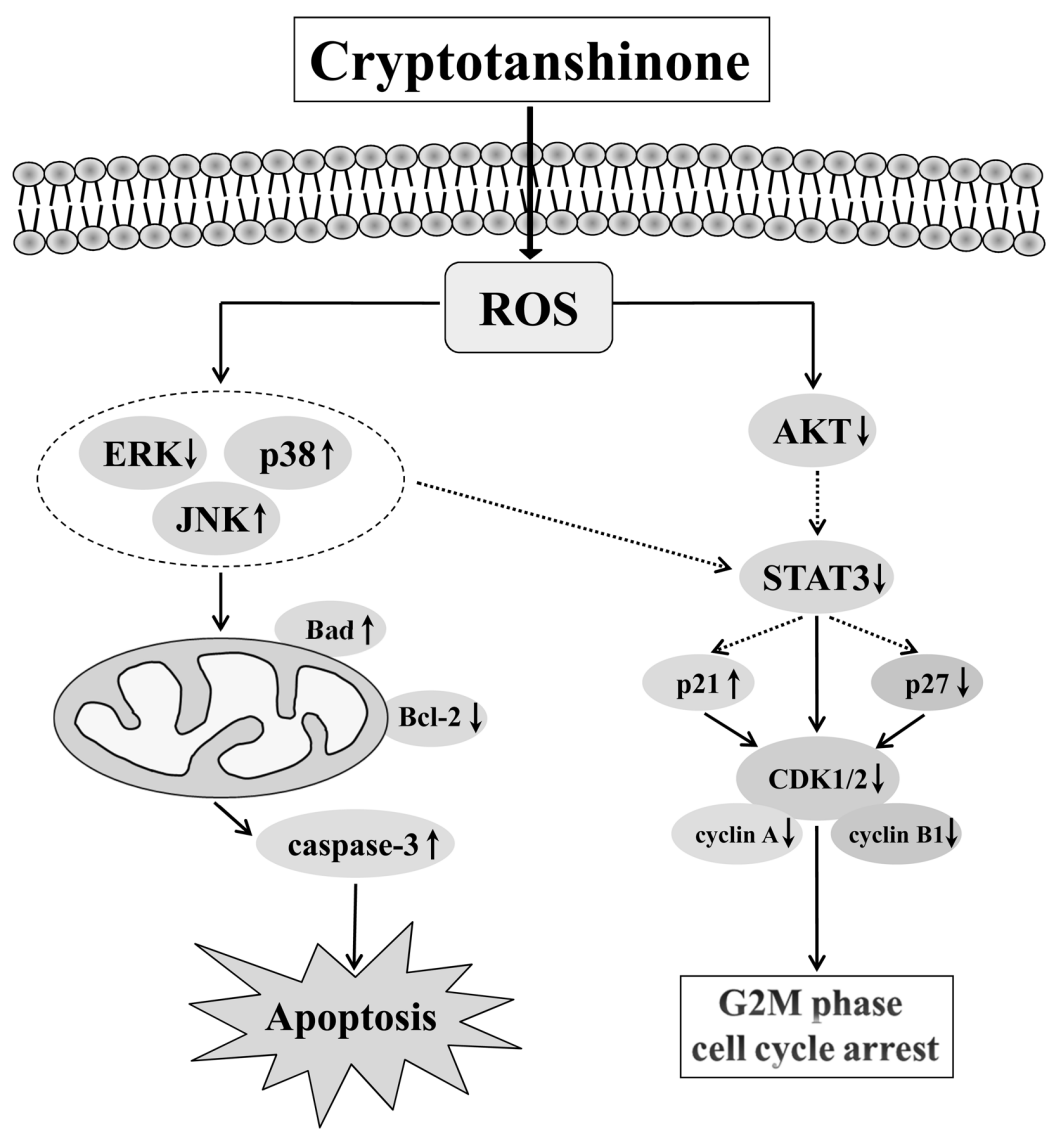
Schematic presentation of the signaling pathways in GC cells affected by CT A proposed mechanism of CT anticancer activity as related to the signal pathways and evidenced by our study.

In summary, the molecular mechanism underlying the action of CT-induced inhibitory activity in human gastric cancer cells (Figure 8) may include ROS generation and subsequently inhibit activation of MAPK and AKT signaling pathways, which increase cell cycle arrest and caspase-dependent apoptosis. All of these ultimately lead to the inhibition of tumor growth. These data strongly suggest the value of further study on the application of CT as a potential treatment for GC.

## MATERIALS AND METHODS

### Chemicals and reagents

CT (Sigma-Aldrich; St. Louis, MO, U.S.) and 5-FU (MedChem Express; Princeton, NJ) was dissolved (20 mM in 100% DMSO; Sigma-Aldrich, St. Louis, MO, U.S.) as a stock solution, and stored at −20°C. This was diluted with cell culture media before use. The final DMSO concentration did not exceed 0.1% throughout the study. All other chemicals were of analytical grade.

### Cell line and cell culture

Human gastric cancer cells and normal liver cells were purchased from American Type Culture Collection (ATCC; Rockville, MD). AGS, MKN-28, MKN-45, KATO-3, SNU-5, SNU-216, SNU-484, SNU-668, L-02 and QSG-7701 cells were cultured in RPMI 1640 medium. NCI-N87, YCC-1, YCC-6 and YCC-16 cells were cultured in DMEM medium. Medium contained 10% heat-inactivated fetal bovine serum (FBS, Gibco; Auckland, NZ), 100 U/mL penicillin and 100 μg/mL streptomycin. Cells were maintained at 37°C in a humidified atmosphere with 5% CO_2_.

### Cell viability assay

The effects of CT on GC cell viability were measured using an MTT assay. Cells (1×10^4^) were seeded in 96-well culture plates, and treated various concentrations (1, 3, 10, 30 and 100 μM) of CT or 5-FU for 24 h. And then, 15 μL of MTT solution (5 mg/mL) was added to each well and incubated at 37°C for 2 h, each well was then removed, and 100 μL DMSO was added to each well to dissolve formazan crystals. Absorption intensity was analyzed using an automatic microplate reader (BioTek Instruments Inc., Winooski, VT) at 490 nm, and results were used to calculate cell viability.

### Cell apoptosis analysis

Annexin V-FITC and propidium iodide (PI) double stain and flow cytometry were used to assay apoptosis. AGS cells were collected after treatment with 10 μM CT at different time points (3, 6, 12 and 24 h). AGS cells were washed with PBS twice and fixed with 195 μL Annexin V binding buffer, followed by the addition of 3 μL Annexin V and 2 μL PI (Annexin V-FITC Apoptosis Detection Kits; Beyotime Institute Biotechnology, Shanghai, China). After incubation for 10 min in the dark, PBS was added to bring the total volume to 500 μL. Then, an EVOS FL Auto Cell Imaging System (Thermo Fisher Scientific, Inc., Waltham, MA) and flow cytometer (Beckman Coulter, Inc., Brea, CA, USA) were used to quantify apoptosis.

### Cell cycle analysis

MKN-28 cells were treated with 10 μM CT at different time points (3, 6, 12 and 24 h). Cells were fixed in 70% cold ethanol at 4°C overnight, then washed with cold PBS and incubated with RNase (10 μg/ml) and PI (10 μg/ml) (Cell Cycle Detection Kits; Beyotime Institute Biotechnology, Shanghai, China) at 37°C for 30 min in the dark. Samples were analyzed with a flow cytometer.

### Measurement of ROS generation

MKN-28 cells were treated with 10 μM CT for different time points (3, 6, 12 and 24 h). Then, cells were collected and suspended in 10 μM DCFH-DA (Merck, Shanghai, China) at 37°C for 30 min. After incubating, cells were washed twice with PBS and resuspended in PBS for ROS accumulation measurement using flow cytometry.

### Western blotting analysis

After treatment with 10 μM CT for 3, 6, 12 and 24 h, AGS cell protein was extracted with cell lysis buffer for 30 min and centrifuged at 12,000 rpm for 30 min at 4°C. Equivalent protein (30 μg) were separated on 8–12% SDS-PAGE and transferred onto a nitrocellulose membrane which was incubated in blocking solution (TBS with 0.05% Tween 20 and 0.5% nonfat milk) for 1 h at room temperature and reacted with primary antibodies (all from Santa Cruz Biotechnology, Inc., Dallas, TX, USA) against mouse monoclonal α-tubulin (1:2,500; cat. no. sc-8035), Bad (1:1,500; cat. no. sc-8044), Bcl-2 (1:1,500; cat. no. sc-7382), caspase-3 (1:1,500; cat. no. sc-373730), p-ERK (1:1,500; cat. no. sc-7383), p-JNK (1:1,500; cat. no. sc-6254), JNK (1:1,500; cat. no. sc-7345), p-p38 (1:1,500; cat. no. sc-7973), p-STAT3 (1:1,500; cat. no. sc-8059), STAT3 (1:1,500; cat. no. sc-8091), p21 (1:2,500; cat. no. sc-397), p27 (1:2,500; cat. no. sc-258), cyclin A (1:2,500; cat. no. sc-751), cyclin B1 (1:2,500; cat. no. sc-245) and CDK1/2 (1:2,500; cat. no. sc-53219). Rabbit polyclonal ERK2 (1:1,500; cat. no. sc-154), p38α/β (1:1,500; cat. no. sc-7194), Akt1/2/3 (1:1,500; cat. no. sc-8312), p-Akt1/2/3 (1:2,500; cat. no. sc-7985-R) overnight at 4°C. Membranes were then incubated with appropriate horseradish peroxidase-conjugated secondary antibodies. Goat anti-mouse IgG (1:5,000; cat. no. ZB-2305) and goat anti-rabbit IgG (1:5,000; cat. no. ZB-2301) were used as secondary antibodies for 2 h, washed with TBST and visualized by using Pierce ECL Western Blotting Substrate (Thermo Fisher Scientific, Inc., Waltham, MA) on AI600 (GE, Fairfield, CT). Proteins were semi-quantified using Image J software.

### Tumor xenograft study

Male 5–6-week-old BALB/c nude mice (18–20 g) purchased from Beijing Vital River Laboratory Animal Technology Company were kept on a 12:12 h light-dark cycle with access to food and water and raised in independent ventilation Cage (IVC). MKN-45 cells (2×10^6^ cells/50 μL) were subcutaneously injected into the flanks of the nude mice at 6–7 weeks-of-age. Once the tumor volume reached 100 mm^3^, mice were randomized into four groups and given: (1) vehicle (DMSO, i.p., once every two days), (2) 5-FU (10 mg/kg, i.p., once every two days), (3) CT (1 mg/kg, i.p., once every two days), (4) CT (10 mg/kg, i.p., once every two days). After 20 days, mice were sacrificed and xenografts were dissected and immediately snap frozen in liquid nitrogen and stored at −80°C until processing. Tumor growth was monitored every two days, and tumor volume (mm^3^) was defined as (l×w^2^)/2, where l is the length and w is the width (mm) of the tumor.

### Hematological assay

On day 20, blood was drawn by puncturing the retro-orbital area of the nude mice in all groups and analyzed using an automatic animal hematology analyzer (Nanjing Perlong Medical, Inc., Beijing, China).

### H&E staining and immunohistochemistry

Organs and tumor tissues from mice were fixed with 10% neutral buffered formalin for 24 h, and then embedded in paraffin, sectioned at a thickness of 5 μm. Slides were stained with hematoxylin and eosin (H&E) followed by dehydration in graded alcohol.

Tumors slides were deparaffinized and rehydrated and antigen was retrieved. After blocking nonspecific protein with BSA, slides were incubated with primary antibodies include p-ERK (1:500), p-JNK (1:500), p-p38 (1:500), p-STAT3 (1:500), and cleaved-caspase-3 (1:500) for 40 min. Horseradish-peroxidase-labeled anti-mouse or rabbit IgG was subjected to DAB staining (Dako North American, Inc., Carpentaria, CA, U.S.) and incubated at room temperature for 30 min. Tissue sections were counterstained with H&E for 40 s. After drying, slides were observed under a light microscope. The relative quantities of p-ERK, p-JNK, p-p38, p-STAT3 and cleaved-caspase-3-positive cells were quantified by counting brown-stained cells within total number of cells in three arbitrarily selected fields.

### Statistical analysis

Statistical analyses were performed according to the Statistical Package for Social Science (SPSS) 19.0. Data are expressed as means ± standard deviation (SD) and analyzed using a one-way ANOVA (*p*<0.05 was considered statistically significantly different).

## Abbreviations

CT: cryptotanshinone
GC: gastric cancer
ROS: reactive oxygen species
NAC: N-acetyl-L-cysteine
JNK: c-Jun N-terminal kinase
ERK: extracellular regulated protein kinases
STAT3: signal transducer and activator of transcription 3
MAPK: mitogen-activated protein kinase
H_2_O_2_: hydrogen peroxide
DMSO: dimethyl sulfoxide
MTT: 3-(4,5-dimethyl-2-thiazolyl)-2,5-diphenyl-2-H-tetrazolium bromide
PI: propidium iodide
Bcl-2: B-cell lymphoma-2
CDKs: cyclin-dependent kinases.
